# Mapping everyday urban political ecologies: Experiential cartography as embodied methodology

**DOI:** 10.1177/30497515251364723

**Published:** 2025-08-08

**Authors:** Philippe Rekacewicz, Tait Mandler, Doms Cordero, Precious Angelique A Echague, Anita Hardon, Bryan Pauchano, Sophia Pelagio, Mariana Rios Sandoval, Michael Lim Tan

**Affiliations:** 1 4508 Knowledge, Technology, and Innovation Group, Wageningen University & Research, Wageningen, The Netherlands; 2 Department of Anthropology, University of the Philippines Diliman, Quezon City, Philippines

**Keywords:** experimental cartography, everyday life, embodiment, urban ecologies, toxicities

## Abstract

Recent years have seen a growing interest in embodied urban political ecology. In this article we offer a generative methodological intervention into the field: engagement with a form of experimental mapping we call experiential cartography. This approach incorporates not only tangible data but also engages the senses, feelings, emotions, and perceptions, offering a richer and more nuanced way of understanding and visualizing the world. To present experiential cartography's potential contributions to UPE research we draw on some recent research in the Philippines emerging from our Embodied Ecologies project that has sought to study and intervene in how people sense, know, and act on the cumulative toxicities that permeate everyday urbanized life. Experiential cartographies of cumulative toxicities offer an interesting case for UPE to explore situated experiences and everyday embodiments of planetary urbanization. We organize our examples around Henri Lefebvre's triadic theoretical formulation of the production of space to argue that experiential cartography involves mapping embodied perceptions of spatial practice, creating embodied conceptions and representations of space, and cultivating embodied spaces of representation based on lived experiences. In this way, experiential cartography provides a methodology for not only (academically) analyzing but also (collectively) intervening into the production of urban spaces and ecologies. We suggest experiential cartography is one way that UPE scholars may pay greater attention to embodied activities, experiences, and knowledges. As the intentions of this article are methodological, we lay out some concrete foundations for UPE scholars (or others) who may be interested in their own experiential mapping practices.

## Introduction

Recent years have seen a growing interest in embodied urban political ecology (Kaika et al., 2023), including integrating the body as a central thematic (Doshi, 2017; Kaika and Ruggerieo, 2024; Mandler, 2022), articulating novel theorizations of the body (Andueza et al., 2020), and incorporating more embodied research methods (Solomon and Kaika, 2024). In support of these calls to bring embodiment into urban political ecology (UPE), we offer in this article a generative methodological intervention into the field: engagement with experiential cartography. Experiential cartographic methods that center embodied, sensory, and emotional spatial knowledge offer UPE a tool to both analyze and transform urban socioenvironmental injustices.

The strength of UPE has long been its heterodox and hybridized lineage (Tzaninis et al., 2020), bringing together Marxist, science and technology studies (STS), feminist, postcolonial and other frameworks as well as approaches from anthropology, geography, urban planning, cultural studies, and elsewhere (see, e.g., edited collections by Heynen et al., 2006; Kaika et al., 2023). Despite numerous influential UPE scholars having backgrounds in the technics of architecture and planning, design, and/or radical geography, there has been little critical engagement with the practice of cartography (although see Brenner and Katsikis, 2020). This is perhaps unsurprising considering cartography's historical role in the production and reproduction of racial capitalist systems of representation and domination over land and bodies (Haraway, 1991), of which UPE is fundamentally critical. Furthermore, like many social science fields and approaches, UPE has generally been more interested in a critique of urban planning practice—for example, demonstrating how it unevenly distributes resources and harms, relies on violent representations, and/or identifying otherwise marginalized political actors (see edited volumes Heynen et al., 2006; Kaika et al., 2023)—than contributing to the articulation of new, potentially transformative, practices.

Experiential cartography has a number of things to offer UPE methodologically. First, we propose that by integrating methods of experiential cartography, UPE can not only generate new critical research about urban socioenvironmental issues but potentially generate new constructive engagements with both dominant and subaltern urban actors, like planners, policymakers, activist groups, and local communities. By engaging in collective mapping, researchers and participants co-produce knowledges and analyses as well as opening up potentials for developing collective actions and interventions, as critical cartographic research, which we discuss further below, has shown (Crampton and Krygier, 2005; Shaadan, 2024). “Placing emotions on maps,” as Caquard and Griffin (2018: 4) put it, “can help urban planners to integrate citizens’ perceptions into the planning process” as well as “be mobilized by marginalized groups and communities to resist unwelcomed development.” Second, these co-produced knowledges, analyses, and creative practices valorize tacit and bodily ways of knowing. This has been called for both in UPE (Loftus, 2009; Doshi, 2017; Solomon and Kaika, 2024) and in the political ecology of toxics literature (Theriault and Kang, 2021) we discuss below. Doshi (2017: 3), for example, has argued that “visceral experiences… [and] affective intensities work through and shape infrastructures and socio-natural flows.” Bodies, she reminds, “are sites for the formation of political subjectivities with sometimes contradictory desires” (Doshi, 2017: 3). Indeed, Stein and Luna (2021) have highlighted precisely such contradictory dynamics of risk and desire in their work on the “toxic sensorium.” Finally, experiential cartography incorporates embodied creative practice into UPE research. Not only do we agree with Solomon and Kaika (2024: 1507) that “embodied research methods developed in arts practices… are especially suited to researching interrelations and entanglements between humans and other-than-humans.” But, following Henri Lefebvre, we suggest there exist an excess in the everyday life of these complex more-than-human entanglements that “can be expressed only through artistic means” (Schmid, 2008: 40).

To present experiential cartography's potential contributions to UPE research we draw on some recent research emerging from our Embodied Ecologies project (Embodied Ecologies Team 2024; Mandler et al., 2025), which has mobilized experiential cartography to study and intervene in how people sense, know, and act on the cumulative toxicities that permeate everyday urbanized life. Our urbanized worlds and lives are awash with (potentially) toxic chemicals that are largely industrially produced. These chemical entities accumulate in bodies and ecologies, not only through spectacular chemical disasters but through the mundane activities of everyday life. Drawing on Lefebvre's (2003: 139) understanding of “industrialization as a step towards urbanization, a moment, an intermediary, an instrument,” we might consider cumulative toxicities—ubiquitous (industrial) toxins that accumulate and add up in unknown ways—as a condition of “planetary urbanization” (Brenner and Schmid, 2015; Lefebvre, 2003). The production, accumulation, and consumption of toxic chemicals are not processes neatly confined to bounded spaces, nor are chemical entities obedient agents that respect molecular, bodily, ecological, or geopolitical boundaries. The innumerable chemicals and unknowable admixtures that, at this point, do not simply permeate but increasingly constitute the activities, relations, and ecologies of human and nonhuman everyday life (Murphy, 2008) are profoundly entangled with planetary networks of infrastructures and activities as well as planetary circulations of materials and bodies that continuously create the conditions for ongoing capital accumulation through the production of space and nature (see Schmid and Topalović, 2023; Smith, 1984).

Our intentions, however, are to explore and map how cumulative toxicities are experienced and navigated in everyday life. To do this we present three examples from our research team in the Philippines. We organize these examples around Henri Lefebvre's (1991) triadic theoretical formulation of the production of space to argue that experiential cartography involves mapping embodied perceptions of spatial practice, creating embodied conceptions and representations of space, and cultivating embodied spaces of representation based on lived experiences. First, in traffic-heavy Metro Manila, Sophia Pelagio mapped embodied perceptions of spatial practice by incorporating the feelings of un/healthiness that accompany her commute through the city. Second, in Marikina's chemical-intensive shoe workshops, P.A. Echague worked with her research participants to create embodied representations of the blurring of their home and work spaces by rendering visible both their feelings in different areas of their home workshops and the diffuse smell of glue. Third, Doms Cordero and Bryan Pauchano cultivated embodied spaces of representation in their research with a community resettled after the Taal volcano eruption by using experiential cartography as a collective process of remembering the past and visualizing possible futures.

We invoke Lefebvre as a way of linking experiential cartography to both a figure foundational to the thinking of much UPE and to recent debates between proponents of empirically and theoretically elucidating planetary urbanization and of empirically and theoretically elucidating situated urban political ecologies (see Tzaninis et al., 2020). Experiential cartography undoubtedly begins with situated, embodied experiences of urbanization—this is perfectly in line with Lefebvre's theory of space, which emphasizes “the materiality of social practice and the central role of the human body” (Schmid, 2008: 40). However, in the process of collectively doing, drawing, discussing, and decoding experiential cartographies, we suggest there exists the potential for opening situated vantages into the planetary. These are not the relatively disembodied—though, in our opinion, no less important—satellite-view cartographies produced by Brenner and Katsikis (2020) of planetary configurations of cables, shipping routes, and hinterlands of the Capitalocene. Instead, they are emotional accounts, textured narratives, of the kaleidoscopic socioecological monstrosity of planetary urbanization as it is immediately experienced in the everyday life of urban denizens. From within the extensive and now over-rehearsed debates in critical geography that are too-often simplified as opposing the planetary and situated, we follow Alex Loftus’ (2018: 93) succinct argument that: “To make sense of planetary accumulation by dispossession means grounding our analyses in a situated understanding of everyday life, while always seeing the praxis of everyday life as internally related to and constitutive of the planetary.” How might scholars engaged in UPE go about researching everyday experiences and situated understandings of cumulative toxicities and other socioenvironmental issues in a way that, as Loftus (2018: 94) insists, “takes seriously the sentiments, hopes and fears” of everyday people? We propose experiential cartography as one methodological tool for doing precisely this.

### Dis/embodied cartographies: Towards experimental cartographies

Traditionally, cartography has claimed the status of an “exact science” based on “reliable data” (Rekacewicz, 2021). It prides itself on providing a neutral and faithful image of reality. However, this approach ignores the political and social use of the map, and its role in both propaganda and protest. Much of the dominant historical practice of cartography has been rightly criticized as relying on a “God-trick” “promising vision from everywhere and nowhere” (Haraway, 1991: 584), and as “a form of knowing or seeing which denies its structuring by the gaze of white male bourgeois knowers” (Pile and Rose, 1992: 131).

There is another way to map and represent the world—an alternative approach. It goes by many names: radical (Cohen and Duggan, 2021), critical (Crampton and Krygier, 2005), alternative, participatory (Plantin, 2014), collective, mental, emotional, sensorial (Perkins and McLean 2020), subjective, and experiential cartography (which includes both emotional and perceptive aspects) (Rekacewicz, 2021). These terms all describe a distinctive mapping practice that extends beyond conventional cartography. This approach incorporates not only tangible data but also engages the senses, feelings, emotions, and perceptions, offering a richer and more nuanced way of understanding and visualizing the world. Despite their varied terminologies, these approaches share fundamentally similar practices.

The literature and practice of critical cartography emerged as a response to traditional cartography's claims to objectivity and neutrality, challenging the notion that maps are purely technical or apolitical representations of space (Harley, 1989; Wood and Fels, 2008). These scholars argue that maps are deeply embedded in power relations, serving as instruments of state control, colonial expansion, and capitalist exploitation (Pickles, 2004; Sparke, 1998). Harley's (1989) deconstruction of cartographic “silences” reveals how marginalized groups and contested histories are often erased from official maps. This critique has been extended by feminist and decolonial scholars who highlight the gendered and racialized dimensions of cartographic knowledge (Radcliffe, 2017). Critical cartography thus repositions maps as contested terrains where dominant spatial narratives can be disrupted and reimagined (Crampton and Krygier, 2005).

In response to these critiques, experimental cartographies have gained traction as participatory, embodied, and often counter-hegemonic practices that challenge dominant spatial representations (Mogel and Bhagat, 2007). These approaches emphasize embodied research practices, where mapping becomes a lived, sensory, and often collaborative process (Kitchin and Dodge, 2007). For instance, feminist scholars have employed walking methods and emotional mapping to document women's experiences of urban space (Springgay and Truman, 2018). Similarly, Indigenous counter-mapping projects reclaim territorial knowledge through participatory methods that center oral histories and non-Western spatial epistemologies (Zaragocin and Caretta, 2020). Such practices highlight situated, relational, and affective ways of knowing spaces and environments.

Recent developments in critical phenomenology bridging phenomenology's focus on lived experience with critiques of power, race, gender, and colonialism (Ahmed, 2006; Weheliye, 2014) have furthered embodied social science research. Scholars argue that perception and bodily existence are never neutral but structured by social hierarchies (Al-Saji, 2014). For example, Sara Ahmed's (2006) work on “orientations” examines how bodies navigate racialized and gendered spaces, while Black feminist phenomenology exposes the hyper-in/visibility of marginalized bodies. This framework has influenced embodied methodologies across disciplines. In cartography, critical phenomenology aligns with experiential mapping practices that center subjective, affective, and politicized spatial relations. For instance, emotional maps of migrant journeys or sensory atlases of urban spaces (Springgay and Truman, 2018) reject top-down cartography by privileging situated, embodied knowledge.

**Figure 1.**
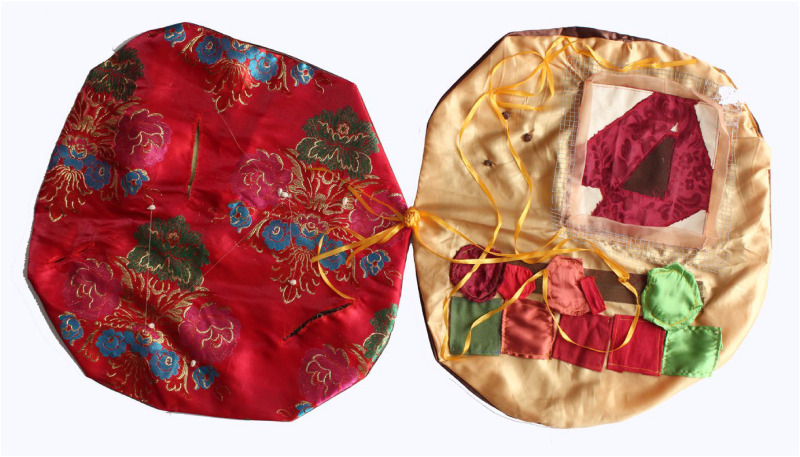
Sensorial mapping of spatial domestic practices of women in Marrakech, Morroco (reproduced with permission from Olmedo, 2011).

**Figure 2.**
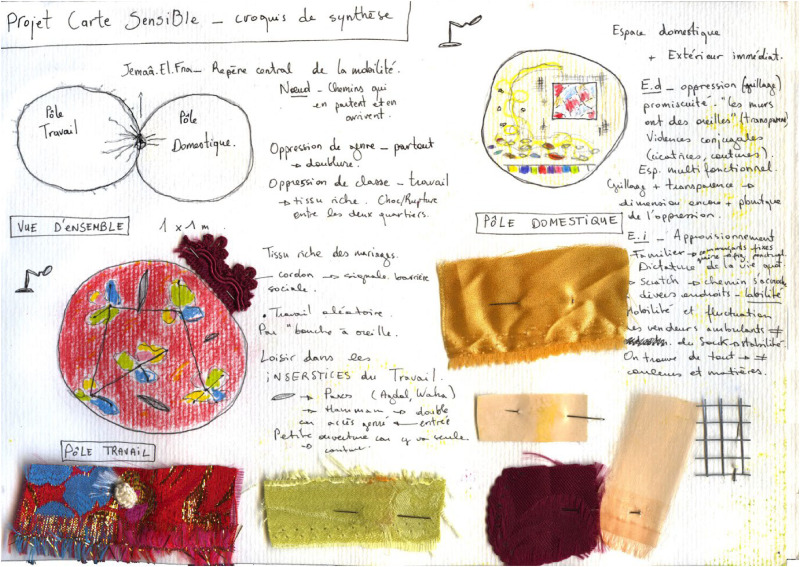
Sensorial mapping of spatial domestic practices of women in Marrakech, Morroco (reproduced with permission from Olmedo, 2011).

**Figure 3.**
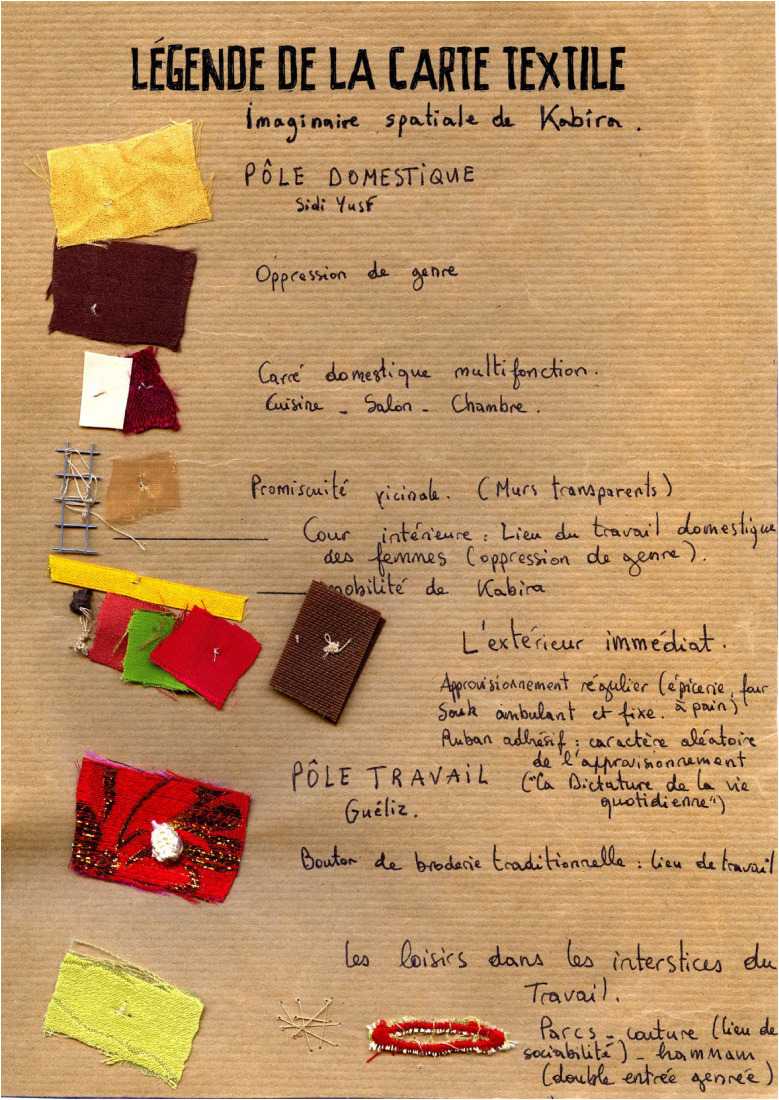
Sensorial mapping of spatial domestic practices of women in Marrakech, Morroco (reproduced with permission from Olmedo, 2011).

Since the early 1980s, these critiques and alternative practices have breathed new air into cartography by proposing different forms of expression. In 2010, Élise Olmedo spent several months living with the women of a working-class neighborhood in Marrakech (Morocco), observing their daily lives and following their movements through domestic spaces and the city (Feildel et al., 2016; Olmedo, 2011). When she attempted to represent these journeys on a map, she realized that traditional cartography offered only a poor and pale reflection of the richness and subtlety of their spatial practices. Conventional maps, unfortunately, fail to convey the emotions that are central to their urban experience. A more flexible mode of representation was needed, one that could align with their perspectives and express the emotions and perceptions tied to the familiar spaces they navigate out of necessity. This led to the creation of a “sensorial map,” an unconventional form of representation that finally captured the “fragrances” of these lived spaces in all dimensions (see Figures 1–3). In this way, Olmedo shows that experimental cartography is truly at the crossroads of art, the human sciences, and activism—in this case, a resolutely sensory form of activism.

Ultimately, experimental mapping practices foreground the political, affective, and embodied dimensions of spatial representation. By centering marginalized voices and experimental methodologies, these approaches challenge hegemonic cartographies while opening new possibilities for spatial justice. When the practice is collective or participatory, it involves the collaboration of a diverse group of people who come together to deliberate and collectively determine how to define and qualify spaces. When it is critical or radical, the focus shifts to deconstructing official or conventional narratives, aiming to create “counter-narratives” that challenge the perspectives imposed by authorities or power institutions. This approach has given rise to the term “counter-cartography,” and practitioners who align with this philosophy often refer to themselves as “counter-cartographers.” Within radical cartography communities—such as the Orangotango + collective and participatory mapping groups in Canada, Germany, France, Chile, and Argentina—the term “counter-cartography” is now debated and challenged (Orangotango+, 2018). It can create confusion, as it may suggest that “counter-cartographers” are entirely opposed to conventional cartographic methods. Their practices are deeply rooted in these longstanding principles. To become a critical or radical cartographer is not to reject or dismantle existing methods to create an entirely new cartographic language. Rather, it is a matter of intent: to deconstruct official approaches and conventional ideas, bringing to light hidden situations, events, or processes that have previously gone unnoticed or overlooked.

**Figure 4.**
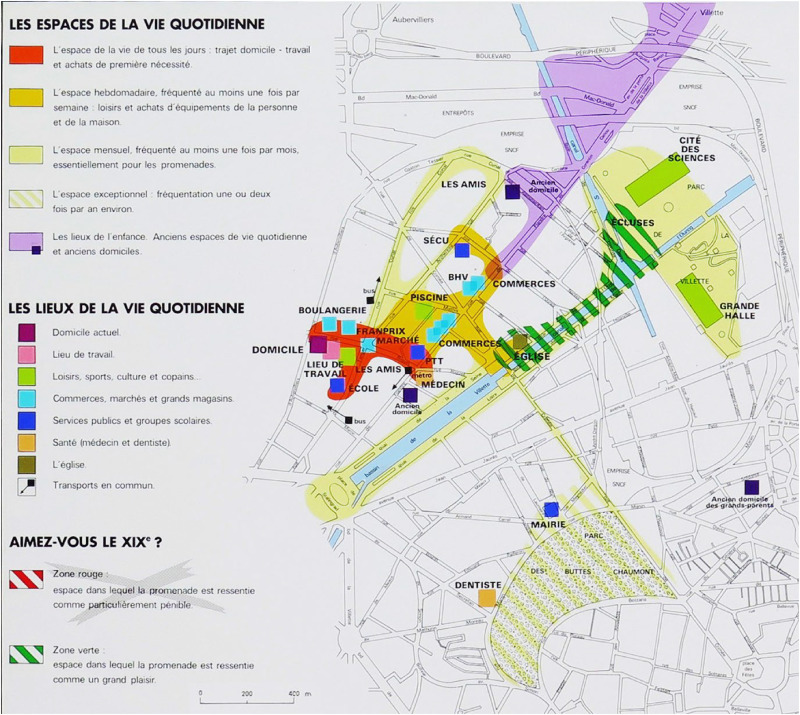
Experiential cartography of the use by the Lenoir Family of XIXe arrondissement in Paris (reproduced from Rekacewicz archives).

**Figure 5.**
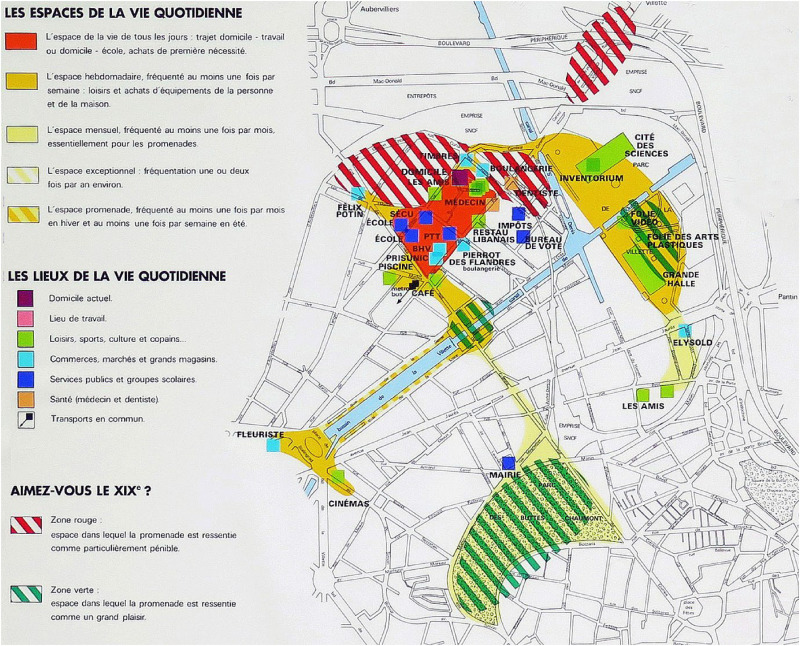
Experiential cartography of the use by the Jordanides Family of XIXe arrondissement in Paris (reproduced from Rekacewicz archives).

**Figure 6.**
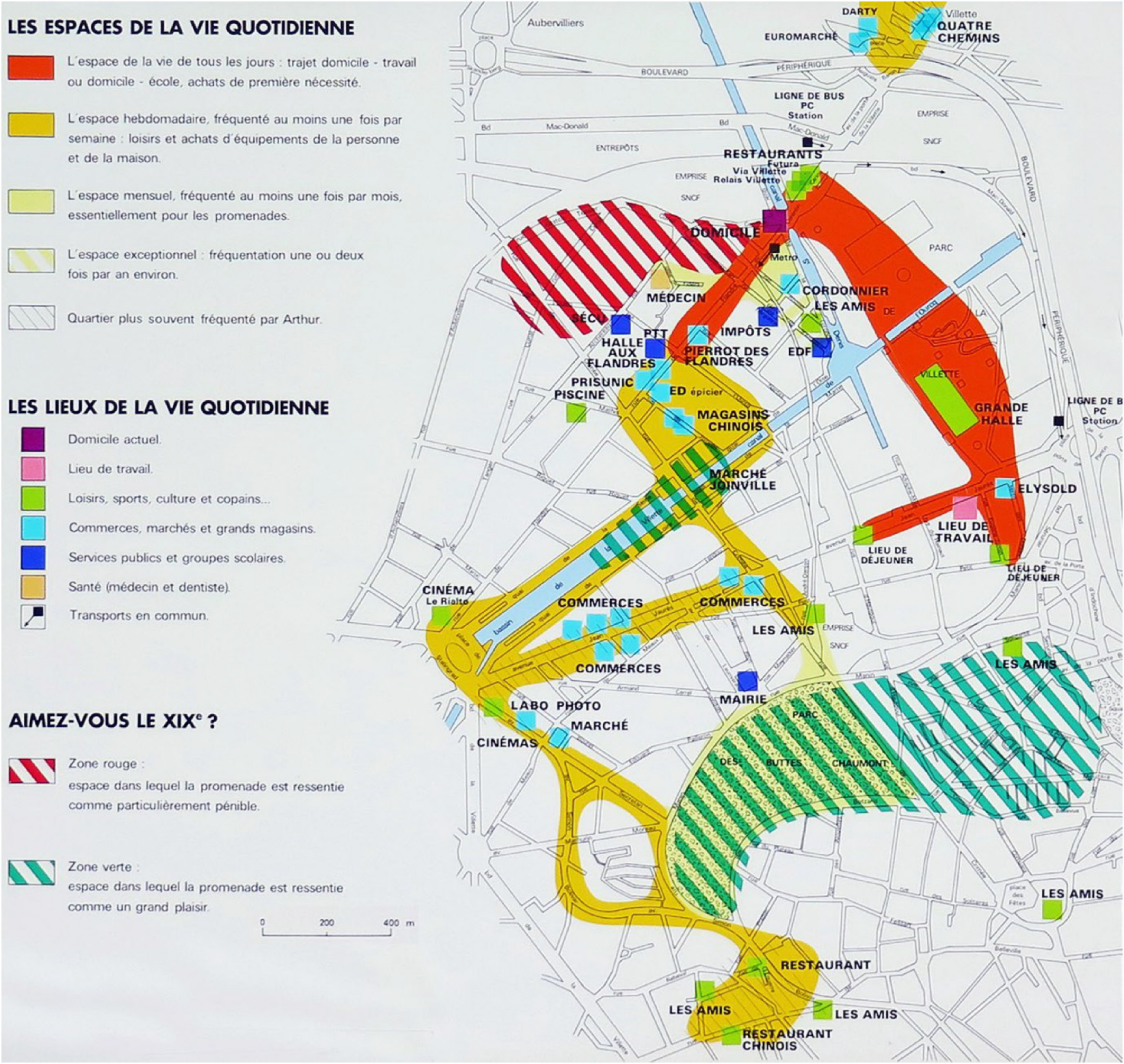
Experiential cartography of the use by the pair Arthur and Amalia of XIXe arrondissement in Paris (reproduced from Rekacewicz archives).

In 1987, the exhibition Les Temps de la Ville at Paris's Cité des sciences, curated by historian Elisabeth Philipp and photographer François-Xavier Bouchart, showcased three experiential maps by Philippe Rekacewicz (Figures 4–6). They explored La Villette's cultural and social memory through residents’ spatial practices, emphasizing time's role in shaping urban experience. The maps traced three families (working-, middle-, and upper-class) over weeks, visualizing their daily movements, preferences, and perceptions of Paris. Combining interviews, walks, and artistic interpretation, the project revealed how social background influenced territorial usage. Unlike traditional maps, these captured subjective, emotional relationships with the city.

Conventional mapping practices have shown us that, in general, human beings and their feelings are often absent or entirely missing from maps. This leaves a significant gap. With experimental mapping, and more specifically experiential mapping, we aim to fill this void and restore “this something.” Experiential mapping helps prevent humans and their emotions from being overshadowed by facts and statistics on a map. In this context, mapping is no longer merely an exercise in representing the world through a strictly ruled (and restrictive) methodology; it becomes a rich fusion of sensory experience, science, geography, politics, and possibly social activism, in which artistic expression is of great importance. Although the method and modus operandi can remain quite structured, the expression must remain entirely free, creative, and artistic.

### Doing experiential cartography: The identity card (Carte d’identité in French), or “the map that says something about oneself”

As the intentions of this article are methodological, in this section we lay out some concrete foundations for UPE scholars (and others!) who may be interested in their own experiential mapping practices. In other words, our goal for this article is not simply leave you with an interest in potentially exploring experiential mapping but instead to actually provide some tools you might use to get started. This experimental cartographic exercise invites participants to create personalized maps based on their lived experiences, memories, and daily practices. By rethinking “cartographic intention,” they can play with or subvert traditional formats, drawing inspiration from artistic approaches. The goal is to produce unique spatial representations—whether maps or other visuals—that reflect personal perceptions of territory, from daily environments to imagined or symbolic places. Participants embed themselves in their maps, tracing life paths, culturally significant sites, and emotional connections to space.

We explore inventive cartographic expression at the intersection of art and social sciences, using an experimental protocol that encourages creative graphic languages. The exercise emphasizes hands-on production with accessible tools, fostering collaboration and exchange. While digital advancements have expanded mapping possibilities (interactive and animated maps), they’ve also led to standardized, impersonal outputs. In response, we turn to experimental and artistic cartography, where intentionality and aesthetics take center stage, offering richer, more subjective ways to depict space.

The goal of the exercise is to familiarize participants with the cartographic creation process using personal and emotional information, with focus on: (1) key elements of life journeys (personal, professional, or family), territorial practices, the way we inhabit and navigate space in the world and (2) sensorial perceptions of events experienced. The invention—or reinvention—of cartographic languages is achieved through a combination of two actions:

*Clear and explicit statements* that enable anyone to learn or reassert the cartographic process and work on the “cartographic intention: What am I representing? How do I structure it? How do I represent it? What do I want to convey? Will the map “speak” to the audience viewing it? Will the map tell a story? Generally, when we look at a map or a visual data representation, we implicitly ask: What is this map telling me? What story does it convey?

*Hand-drawing work*: As it is a reflexive process, everything can preferably be done by hand, on white or colored paper in A4 or A3 format, optionally with base lightly printed geographical base maps, and all the necessary drawing materials (black pencils, colored pencils, an eraser, rulers, squares, fine or thick markers, oil pastels, paint, scissors, tape, glue, craft knives, cardboard, magazines and newspapers, textiles, wool, sewing and knitting materials, small wire for participants who wish to create collages or special artistic installations), as well as reproduction equipment (color printer and scanner). But for those who want, it is also possible to use the computer and drawing software or graphics processing.

This exercise is inspired by research on cartography and sensorial territories initiated in 2008 (the first workshop was held in Amsterdam in May 2008 at the “Netherlands Foundation for Visual Arts, Design and Architecture”). It is designed for all disciplines (geography, sociology, environmental psychology, anthropology, design, art, architecture, urban planning, etc.) as well as individuals outside the academic sphere (NGOs, artists, engaged citizens, etc.). The principle of the exercise is to allow participants to fully immerse themselves in the map—an activity in which they will be invited to project themselves, find their “places of life,” and visually express the emotional and sensitive aspects of geographic situations. In other words, the goal is to incorporate “feelings” into the map alongside “factual” information. The exercise is a participatory encounter, conceived as a moment of collective learning. In practice, this involves meaningful exchanges among participants as well as between participants and the facilitators or coordinators.

#### Methodological Procedure: A Five-Step Protocol

We propose here a method for creating the map in five steps. We could certainly suggest other breakdowns and propose production protocols in just three or four phases, but to reassure users who are not necessarily familiar with cartographic creation, a slightly more detailed approach would allow—during production—to “latch on” to an identified step if apprentice producers were to get lost along the way. These steps unfold as follows:

- **Step 1:** Defining the intention;
- **Step 2:** Collecting and processing data and information;
- **Step 3:** Roughly sketching ideas emerging from the processing, in other words, building the architecture (or the skeleton) of the map;
- **Step 4:** Applying semiology and geometry principles; and
- **Step 5:** Finalizing the artwork into a composition, an assemblage.

We invite participants, throughout the production process, to think (beyond organizing information to make it accessible and understandable to the public) about the artistic means to be implemented (physical structure, dynamic movements, harmony of forms, and blending of colors). From a methodological perspective, participants begin the exercise by developing a “cartographic intention,” followed by collecting and reflecting on relevant information and data. They then transition from mental conceptualization to drawing, after being introduced to a few essential principles of graphic semiology.

**Figure 7.**
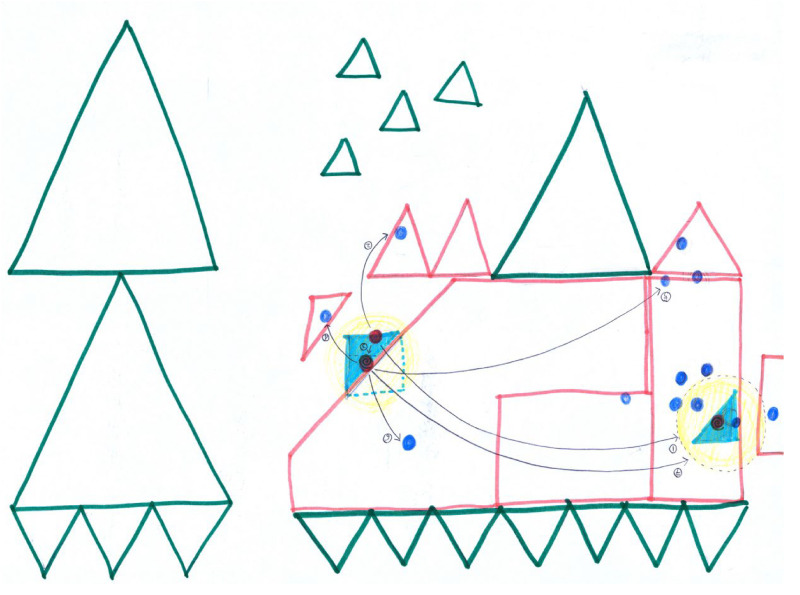
“My world footprint” by Na Kim, Korean artist in Amsterdam, May 2007 (reproduced from Rekacewicz archives with permission from the artist).

**Figure 8.**
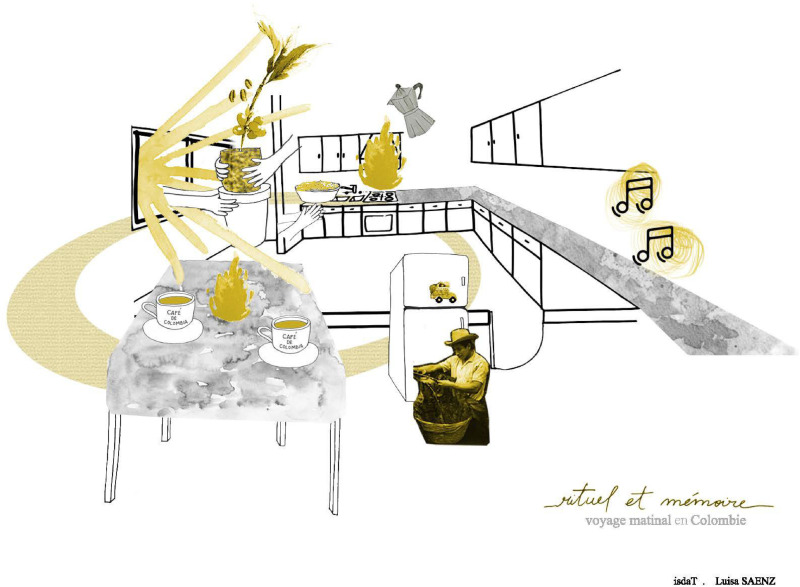
“Emotional and sensorial map of my kitchen” by Luiza Saens in Toulouse, November 2020 (reproduced from Rekacewicz archives with permission from the artist).

This exercise can be conducted on various scales, ranging from a global level (world map—Figure 7) to national (country map), regional (district or departmental map), local scales (exploring how individuals project themselves within a neighborhood or across different areas of a city), and eventually at the large scale of an apartment or a house, if not a room (Figure 8).

For an example of the global scale: In 2007, a radical cartography workshop held in Amsterdam led to the production of a series of global-scale maps, each showing the life footprint, as well as the social and cultural imprint of the participants. Na Kim, a Korean visual artist and graphic designer, envisioned her world—the world where she had left her marks and traces—in a reversed representation and an articulation of her movements and the places significant to her cultural and social identity. This was depicted through a semiological and geometric ensemble (points and lines on a surface) that was very well thought out, elegant, and seamlessly continuous (Figure 7).

For an example of the local scale: In 2020, students from the art school of Toulouse spent a week drawing the sensorial and emotional map of their kitchen, trying to show how they were using it and why this room was so important and meaningful. Luiza Saenz explored the morning use of her kitchen in France is an opportunity for a symbolic journey to her native country, Colombia (Figure 8). It's worth noting that the “practice and use” of the kitchen was admirably conceptualized and depicted by the Swedish movie director Bent Hamer's film, “Kitchen Stories” *(Salmer fra kjøkkenet)* in 2003.

The cartographic representations produced figurative interpretation of territories, enabling us to extend our research and reflections on how to represent territories—not merely in a descriptive manner, detailing everything visible and present, but also, and more importantly, in inventing ways to represent sensitive, uncertain, and ambiguous information, as well as movements that are sometimes invisible and not easily detected even by experienced observers. For this reason, we have experimented with experiential cartography in our research into cumulative toxicities in everyday life as these exposures are structured by dynamics of in/visibility that may be interrupted by the incorporation of cartographic and artistic methods. In the following section we explain the issue of cumulative toxicities and why embodied research methods are called for before turning to some examples from our research.

### Embodied ecologies of toxic worlds

Alongside scholars concerned with “a permanently polluted world” (Liboiron et al., 2018), “a new age of toxicity” (Walker, 2011), and “toxic modernity” (Grandia, 2021), we consider what we call cumulative toxicities an enduring socioecological condition permeating our increasingly urbanized everyday lives and environments (Mandler et al., 2025). Understanding cumulative toxicities as a socioecological condition means considering their emergence from intimate, historical, and ongoing entanglements of entities, systems, and processes that are inseparably chemical, social, and ecological. It also means considering cumulative toxicities not as a discrete problem that exists somewhere “out there” but as a mundane reality that is widely shared—although its harms are profoundly unevenly distributed—and viscerally embodied in the everyday—although the social, economic, and technoscientific systems that undergird these toxicities transcend scales from the molecular to the planetary.

There is a significant body of literature that Theriault and Kang (2021: 6) helpfully review and describe as “political ecologies of toxics,” within which they include “any attempt to understand how social structures, cultural differences, and power dynamics shape the production, distribution, conceptualization, and embodiment of industrial toxics generated at any point in the capitalist world system.” As they argue, alongside others, studying cumulative toxicities raises a number of methodological questions. First, “toxics are substances that do bodies harm, and thus toxicity is inherently relational. Yet conventional approaches to toxicology, epidemiology, and environmental health tend to conceive of both substances and bodies in isolation” (Theriault and Kang, 2021: 6). However, research in toxicology increasingly points to processes of bioaccumulation and biomagnification through which long-term exposure, even at low doses, is linked to various negative health outcomes for boths bodies and ecologies (Wang et al., 2020). In other words, classic approaches in toxicology are inadequate for grasping the way chemicals accumulate and persist. This has led a number of social science researchers to identify and valorize different ways of knowing (Balayannis and Garnett, 2020; Fiske, 2018; Fortun, 2012), emphasizing, for example, the importance of “bodily knowledges” (Shapiro, 2015) and “canary science” (Grandia, 2021). At the same time, of course, cumulative toxicities may often be characterized by their imperceptibility, and as many researchers have shown a “politics of invisibility” (Davies, 2022) is often part of maintaining toxic ecologies. Still, Davies (2022: 419) warns of too-quickly casting exposed communities as “unsighted” as there are “many mechanisms, embodiments, and formations of informal knowledge that allow communities to recognize and live with pollution.” Wiebe (2016), Liboiron (2021), and others also insist on the importance of indigenous ways of knowing that incorporate corporeal experiences and other forms of knowledge.

While the bringing together of different epistemologies is undoubtably as full of pitfalls as potentials, attempting to knit frictious collages of various ways of knowing is part of the necessary leap into the unknown of creative and transdisciplinary research. For example, much has been made of the disjunctures between marxian and indigenous ways of knowing, however the idea, as Glen Sean Coulthard (2014) writes in *Red Skins, White Masks,* is to let Marxism “be transformed in conversation with the critical thought and practices of Indigenous peoples themselves.” One way through such “uneasy alliances” (Benhabib, 1995) is to ground research and analysis first in situated everyday experiences, as Paulo Freire (2015) does in *Pedagogy of the Oppressed*. Just as Freire looks to develop understandings of “domination” built through gathering and decoding lay epistemologies, “lay epidemiology” (Watterson 1994) has been central to developing understandings of toxicity as “public knowledge of community toxic hazards… has largely stemmed from the observations of ordinary people” (Brown and Mikkelsen, 1997: 127).

This brings us to a final methodological concern: what Tuck (2009: 409) calls “damaged based research” which “documents people's pain and brokenness to hold those accountable for their oppression” but that “simultaneously reinforces and reinscribes a one-dimensional notion of these people as depleted, ruined and hopeless.” Building on Deleuze and Guattari (1987) theorization of desire as an assemblage, which is made of distinct experiences that together form a coherent, yet dynamic whole, Tuck offers desire-based research as an antidote to the reductive focus on damage. This methodological move helps attend to complexity and contradiction in human experience, as desire can “reach for contrasting realities, even simultaneously” (Tuck, 2009: 418). Likewise, embodied experiences of toxicity exceeds that those of toxic damage, as Stein and Luna (2021) explore through the notion of the “toxic sensorium,” which involves both the felt recognition of chemicals and the messy dynamics in which being aware of risks and desiring chemicals anyway overlap. For these reasons, much of the political ecology of toxics literature proposes new, creative, collaborative methods. For example, Theriault and Kang (2021: 19) “urge political ecologists to embrace this work with inclusive methods, promiscuous contacts, rigorous ethics, and active imaginations.”

The Embodied Ecologies project is a collaborative inquiry into how people sense, know, and act to reduce chemical exposures in everyday urban life (see Embodied Ecologies Team, 2024; Mandler et al., 2025 for an extended description). It explores the interconnectedness of bodies and ecologies through chemical exposures. The project is multisited, with fieldwork being conducted in the Philippines, France, and the Netherlands, and considers the issue of cumulative toxicities across multiple scales, from the individual to the planetary. Since cumulative exposures emerge, by definition, across space and time, our approach works with ethnographic, historical, and cartographic methods. We offer mapping as a fruitful method for investigating and transforming the dynamics of in/visibility that are central to the epistemological and political problem of cumulative toxicities. Through various practices and forms of participatory, collaborative, and experiential mapping, the embodied knowledge of our interlocutors can be made visible and tangible. Mapping can also connect sensorial experiences with political, economic, social, and regulatory forces that shape uneven exposures but play out over larger histories and geographies that elude everyday perception.

We will present some of the mapping work that has been carried out in the Philippines over the past 3 years. As an international project funded by the European Research Council and housed at Wageningen University and Research in the Netherlands, one way we are trying to navigate the uneven power-geometries of funding flows, data collection, and academic publishing between institutions and researchers in Europe and the Philippines is to support research assistants in publishing under their own name. Accordingly, the research we use for examples here has already been published by the researchers themselves on the open-access cartographically-inclined platform *visionscarto.net* (operated by a collective that includes co-author Philippe Rekacewicz).

### Experiential cartographies of everyday toxic ecologies

To survey how experiential cartography might methodologically contribute to UPE research, we present some preliminary fieldwork done by Embodied Ecologies researchers in the Philippines that explored: feelings of un/healthiness moving through traffic-heavy Metro Manila (Pelagio, 2025), the blurring of work and home spaces in Marikina's chemical-intensive shoe workshops (Echague, 2025), and the experiences of communities resettled after the Taal volcano eruption (Cordero, 2025; Pauchano, 2025). In each case we find people grappling with and navigating processes of urbanization entangled with various toxicities (such as exhaust, glues, and ash) in their everyday lives. Some seek to reduce their exposures while others downplay the negative health effects, still others try to remember what life was like before a “natural” disaster. As a methodology, experiential cartography opens up potentials for embodied politics of urban ecologies: first, as a collective practice engaged in the encoding and decoding of representations (there are similarities here with Freire's [2015] *Pedagogy of the Oppressed*); and second, as a creative practice of poesis. Here we find ourselves aligned with part of Henri Lefebvre's political-philosophical project to “extend the boundaries of artistic practice into the quotidian, thereby demonstrating its role in revolutionary change… Lefebvre places emphasis on the role of creative action in the struggle for a future society” (Loftus, 2012: *xx*). Inspired by his emphasis on “the creative, poetic act” and the significance of his thinking for much UPE scholarship, we use Lefebvre's (1991) triadic theorization of the production of space to organize our three examples.

Lefebvre's writing on the production of space has proven to be a near-endless fountain of both inspiration and confusion for geographers and all manner of other scholars interested in space. Our purpose here is not to present a detailed reconstruction or explanation of Lefebvre's theoretical formulations—for that we will rely extensively on Christian Schmid's (2008; 2023) work. Lefebvre argues that “space is to be understood in an active sense as an intricate web of relationships that is continuously produced and reproduced” (Schmid, 2008: 41) through the dialectical interplay of three interlinked dimensions, which he viewed in two ways. First, linguistically, as: spatial practice, representations of space, and spaces of representation. Second, phenomenologically, as: perceived space, conceived space, and lived space. Importantly, these are not separate or distinct spaces, but interrelated dimensions of the production of space.

Our idea is that the poesis of experiential cartography is a methodology that involves simultaneously mapping embodied spatial practice/how everyday ecologies are perceived, creating embodied representations of space/alternative conceptions of everyday ecologies, and cultivating embodied spaces of representation/new meanings and spatial symbols from everyday life.

#### Mapping embodied perceptions of spatial practice

For Lefebvre, spatial practice designates “the material dimension of social activity and interaction… In concrete terms, one could think of networks of interaction and communication as they arise in everyday life (e.g., daily connection of residence and workplace) or in the production process (production and exchange relations)” (Schmid, 2008: 36). The phenomenological version of this is what he called “perceived space,” which “comprises everything that presents itself to the senses; not only seeing but hearing, smelling, touching, tasting. This sensuously perceptible aspect of space directly relates to the materiality of the “elements” that constitute “space” (Schmid, 2008: 39). Experiential cartography maps the materiality of spatial practice through attending to its perception by a particular person or group of people. Compared to other cartographic practices, it elucidates how people's embodied experiences and tacit knowledges are intwined with social activity and its material basis. The ‘urban fabric’, as Lefebvre liked to call it, is perceptible and how people perceive it shapes the very same everyday activities and movements that contribute to its production. Experiential cartography not only takes these perceptions as seriously as property lines are taken seriously on a cadastral map, but as a practice it offers a creative way for participants to reflect on their own perceptions of the existing urban fabric, how these perceptions shape their engagement with it, and what kind of other urban fabric they might prefer.

One of our preliminary experiments with experiential cartography was asking people to map and narrate their everyday itineraries. In the Philippines, Sophia Pelagio mapped her everyday life, her urban itinerary, how she felt as she moves across the city, and where she feels good and bad (see Pelagio, 2025). After Sophia, along with a number of other participants, conducted the mapping exercise on their own, including taking pictures, videos, and voice notes of their everyday urban movements, we came together for a collective discussion—or decoding—of their maps and experiences. At this early stage of our project, we were less interested in data collection than exploring the methodology and learning what kinds of information and stories may emerge.

**Figure 9.**
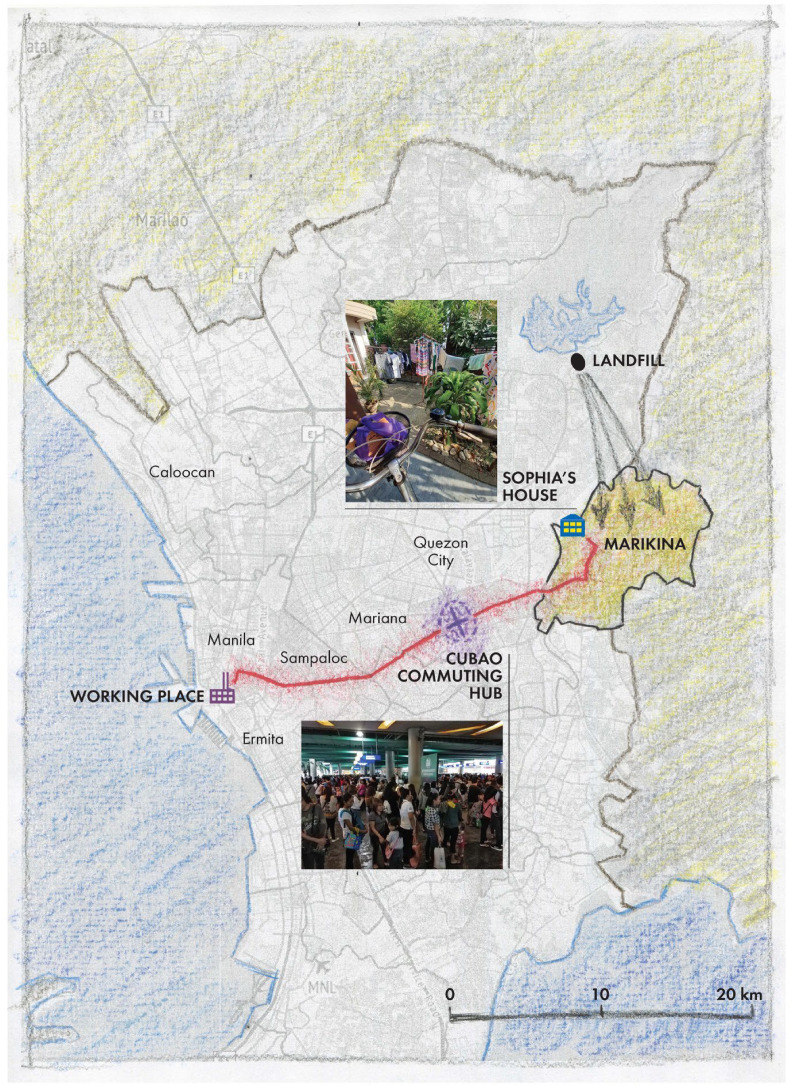
“Commuting from home to work” by Sophia Pelagio (2025), redrawn by Rekacewicz.

Along with other participants in Manila, Sophia identified commuting hubs as among the most unpleasant and toxic places (Figure 9): “I consider the terminals very toxic because oftentimes I would wait in line for about 30 to 45 minutes. These public utility vehicles, especially the jeepneys, would not turn off their machine, their engine. So, all the toxic gases we would tend to inhale.” While waiting, moreover, many people smoke, making the air even more dense. Suffocating was the word used to describe her daily experience. In our group discussion afterwards, we began to tease out some of the power-laden socioecological relations underlying the toxicity of commuting: “There are class connotations to commuting. If you commute, it means you take public transport—not one ride, but several as you are likely to live far from your place of work. And then there's a hierarchy. There are jeepneys, light rail, and all… I think it's one of the reflections of the failure of the state. The inability to provide clean, safe, rapid public transport and quality of life is so bad because of that commute… It's during the waiting that you have a lot of the embodying we’re investigating. The sweaty bodies, the smells in the environment. They become so much more concentrated because of that horrible commute.” Indeed, cumulative exposures to toxic chemicals are embedded in the “interlinking chain[s] or network[s] of activities or interactions which on their part rest upon a determinate material basis (morphology, built environment)” (Schmid, 2008: 37).

This kind of creative cartography works as a technique for researchers and participants to reflect on their everyday lives, express how their inner emotional world is mediated by and maps onto their urban surroundings, and thus begin considering the trans-scalar entwinements of embodied ecologies. Sophia's mapping exercise also made visible how toxicities accumulate across time and space as bodies move through entanglements of urban spatial practices. This offers a different understanding of toxicity, as Shadaan (2024: 204) explains: “Redefined, the body burden involves the bodily accumulation of various types of harms that result from exploitative, oppressive, and otherwise harmful systemic and structural conditions. This approach is distinct from toxicological definitions, which reduce the body burden to chemical toxicants—a molecular-level hyperfocus that can obscure the broader context of exposure.” In Sophia's experiential mapping of her emobided perceptions of space, we begin to see how both the exposure and experience of toxicities, like cigarettes or exhaust fumes, emerge through where, how, and why bodies move through space. In other words, the class-structured spatial practice of commuting is as internal to the toxicities of exhaust fumes as is carbon monoxide—neither chemical nor context take inherent precedence.

#### Creating embodied representations of space

Representations of space “give an image and thus also define a space… Lefebvre counts maps and plans, information in pictures, and signs among representations of space” (Schmid, 2008: 36). Experiential cartography obviously intervenes in this aspect of the trialectic, turning embodied perceptions of spatial practice into representations that differ from dominant cartographic expressions by interlayering sensations and feelings with other information. For Lefebvre, the phenomenological aspect here is what he calls “conceived space,” which involves “bringing together the elements to form a ‘whole’” such that what is “considered or denoted as space presumes an act of thought that is linked to the production of knowledge” (Schmid, 2008: 39).

**Figure 10.**
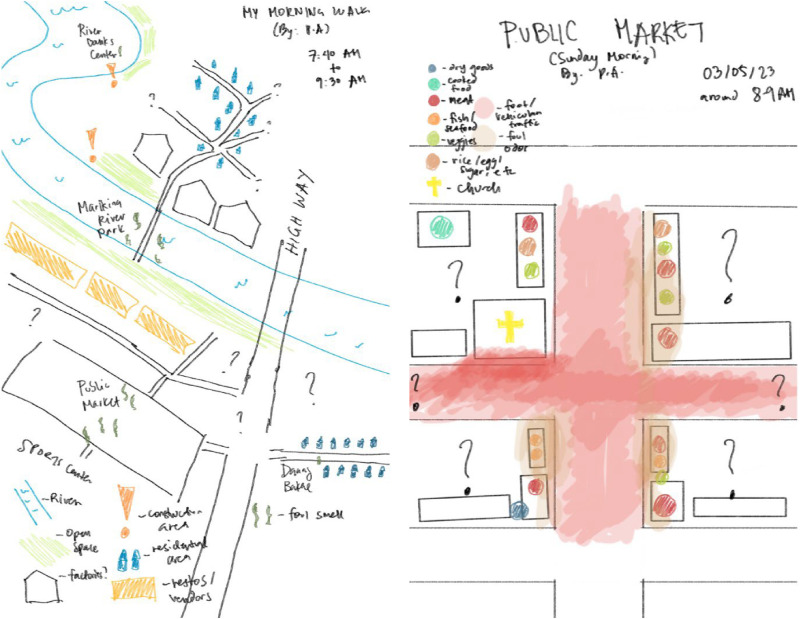
“Preliminary experiential maps of initial pagawaan visits” by Echague (2025).

In Marikina City, an area within Metro Manila, there is an alleyway that has been home to small-scale shoe workshops (pagawaan) for multiple generations. P.A. Echague conducted research with the people who not only work but live in this alleyway, as the pagawaan is generally located below or alongside the shoemakers’ houses (see Echague, 2025). P.A.'s research was focused on shoemakers’ exposure to industrial-purpose glue which contains chemicals classified as “dangerous” in the Philippines and have been linked to health problems. The glue is used to make shoes in the pagawaan, in which working and living space is not only proximate but blurred through the movements of people, air, smells, and more. P.A. began her research with her own experiential mapping of the alleyway and of some of the pagawaan (Figure 10).

P.A. began mapping by sensing. In her fieldwork, the intense smell of the glue struck her the most, overriding her other senses. She wanted to translate this into maps and highlight the strong smell of the fumes of the glue, as experienced by her body. Paying attention to the different kinds of smells present in the alleyway and the pagawaan, she tried to illustrate her experience through colors. She particularly used crayons, although digitized, to try and show the overlapping scents present in the pagawaan, not only from the glue, but other harmful materials like synthetic leather. These visualizations, however, do not only show what kinds of materials are used by shoemakers, but also show how these different toxicants permeate to the alley, and into their homes.

**Figure 11.**
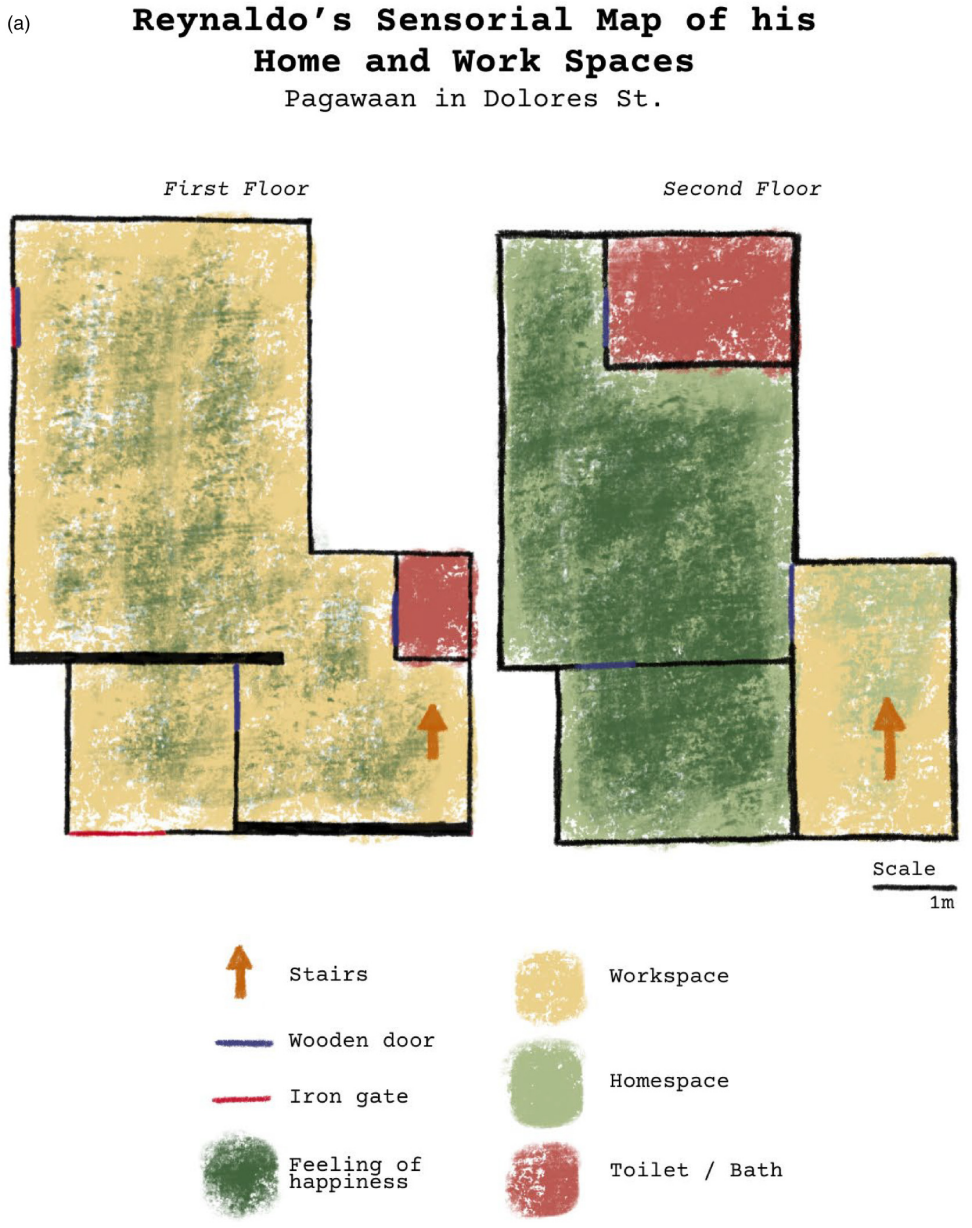

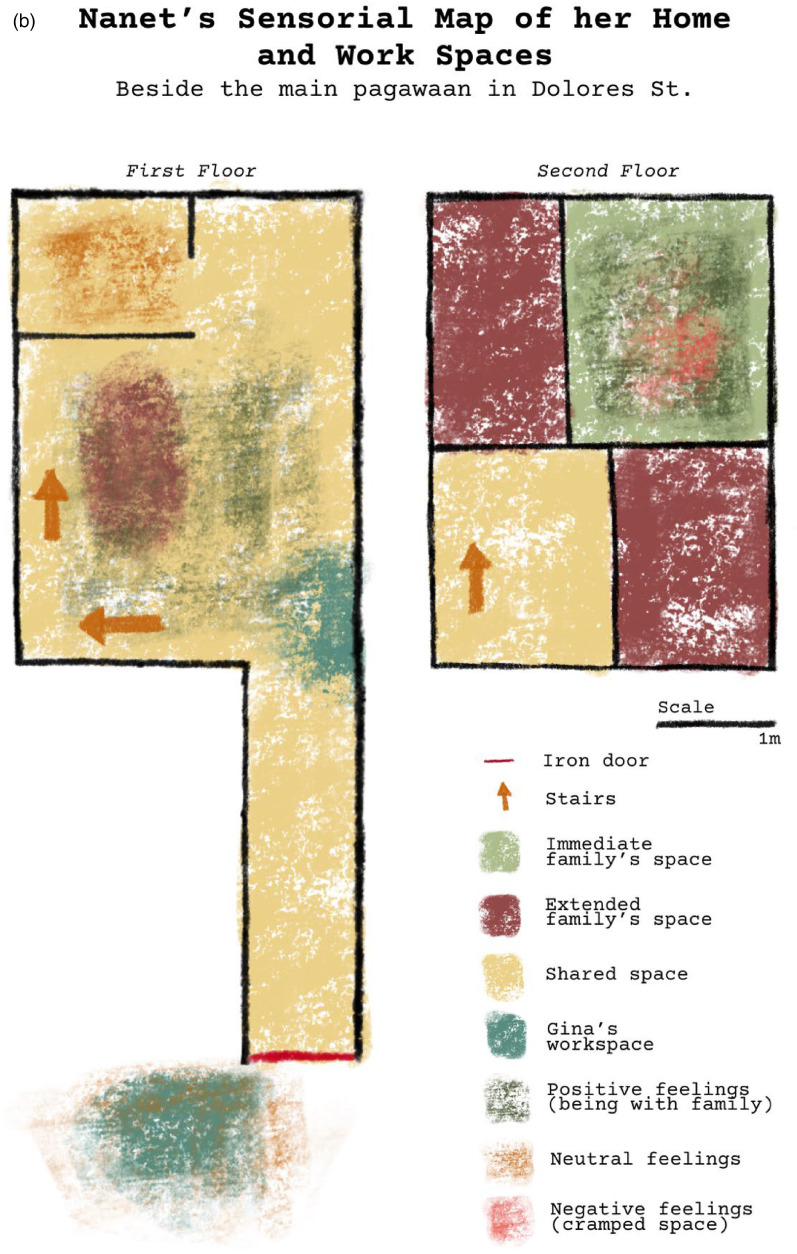
(a) and (b) Nanet's and Reynaldo's experiential maps, postprocessed by Echague (2025).

P.A. also did experiential mapping with her research participants, asking not only where different activities are done but how they feel about different parts of their living-working spaces. Through this, research participants were able to describe what they feel about certain parts of the alley and also assign symbols in the form of emoticons, that is, smiley and sad faces, to represent their experience of the space. P.A. then translated these symbols into overlapping colors, to represent the overlapping negative and positive feelings the research partners have about their living and workspaces (Figure 11(a) and (b)).

In her mapping of the alleyway as a lived space, she prioritized the activities that go on over the precise morphology of the built environment, producing narratives that were initially invisible to her. By mapping scents and her research partners’ feelings of the space, she highlighted how shoemaking and the chemicals used in the industry are part of the community's everyday life. And by further probing into their feelings of the space, the maps and interviews reveal other forms of toxicities experienced by her research partners. Other than harmful chemicals and materials used in the pagawaan, her research partners noted having very cramped homes, being regularly affected by flooding, smelling stench coming from sewers outside their homes, and even working longer hours with toxicants to provide for their families.

Through experiential cartography, she came to understood how her partner community is subject to different forms of toxicities on a daily basis, and how these are interlinked with structural injustices and power-geometries, from urban planning practices (or lack thereof) to international markets that demand certain production practices to compete—such as reducing labor costs by working with informal contracts between neighbors and using more toxic glue that creates higher quality shoes. In other words, as Shadaan (2024: 199) writes, “body and hazard maps tell us about more than embodied and workplace-situated harms; they show the material impacts of broader scales of violence.” What P.A. heard from many of her participants is that they are “sanay” to the toxic glue, meaning, in Tagalog, that they are “used to it.” P.A.'s experiential maps then not only make visible the spatiosensoriality of toxic glue but the contradictory dynamics in which shoe-makers can feel happiness in their workspaces where the smell of glue is strong. In other words, cumulative toxicities occur and are experienced in complex and contradictory ways that creating embodied representations of space can help bring to the fore.

#### Cultivating embodied spaces of representation

For Lefebvre, spaces of representation refers to “the process of signification that links itself to a (material) symbol. The symbols of space could be taken from nature, such as trees or prominent topographical formations; or they could be artifacts, buildings, and monuments; they could also develop out of a combination of both, for example as ‘landscapes’” (Schmid, 2008: 37). This is also understood as “lived space,” which “denotes the world as it is experienced by human beings in the practice of their everyday life. On this point Lefebvre is unequivocal: the lived, practical experience does not let itself be exhausted through theoretical analysis. There always remains a surplus, a remainder, an inexpressible and unanalysable but most valuable residue that can be expressed only through artistic means” (Schmid, 2008: 40). The practice of doing experiential cartography, especially as a collective endeavor, is a process of signification in which participants appropriate and create symbols of space to express their emotional lives through artistic means.

In January 2020, the Taal volcano, located in the south of Luzon about 60km south of Manila, on an island in the middle of a lake, erupted, spewing a massive amount of gas and ash that settled into a layer several meters thick. Several hundred residents from the island evacuated, relocating to the municipality of Talisay, which faces the volcano island. A portion of this community found refuge in a neighborhood built in Tranca, about 10km from the lake's shore. Social and cultural life was reorganized as best as possible within the community, whose members had lost everything. Two anthropologists, Doms Cordero and Bryan Pauchano, took an interest in this community and initiated a dialogue with its members to understand how they were adapting to their new living conditions (see Cordero, 2025; Pauchano, 2025). They invited participants from the *pabahay* (or resettlement area)—mostly women—to participate in an experiential mapping activity. The goal of this cartographic exercise was to visualize their “life before,” a memory of how and where they lived preceding the fateful eruption.

The five participating women, armed with colored pencils and markers, gathered around a large one-meter-by-one-meter map of the island, placed on a table in the middle of one of the refugee housing units. There were initial hesitations encountered, including when participants found it difficult to try and make out what they were seeing from the makeshift, grayscale print of the island. The participants were also shy, admitting that they did not often draw and color. But soon enough, the atmosphere turned joyful, with lively discussions as they recalled memories that progressively took shape on the map: guiding tourists on horseback, common meals, profitable small businesses, and going out with girlfriends. The map began to take on colors and forms!

After an hour of continuous drawing and conversation, their expressions grew more serious, and the discussions became more somber. It was as if recalling these happy memories had plunged them into nostalgia for a beautiful life now obliterated, gone. While the energy around the room remained light, it was equally important for the researchers to listen with intent and sincerity. The stories shared were more than just mere recollections—they were memories imbibed with various emotions and aspirations that relayed what they truly held meaningful in their lives.

Ultimately, in this initiative, the most interesting result was not the map itself—showing only frequented places and pathways—but rather the undirected effect the map brought to them. The process of creating the map allowed these women to reflect on their current status as refugees compared to their previous lives. It was a stark awareness of the deterioration of their condition as women, now entirely dependent on government aid and especially on their husbands, who were more often than not the only ones able to retain their employment status. Through the mapping activity, stories of the island as a source of *kabuhayan* (livelihood) for its residents elaborated on the intimate, nourishing relationship the community had with the land. This was in sharp contrast to their much more independent lives on the island, where they worked, earned a good living, and had a certain level of autonomy within their relationships and families. But while evoking these memories may remind them of the grave disparities they faced 5 years ago, they also serve as markers for their continued aspiration for a better life.

This participative approach in cartography was able to bring community members together by revisiting a part of their lives buried under the literal and metaphorical ash of disaster. It was an unexpectedly heartwarming emotional activity that allowed everyone in the room to catch a glimpse of what used to be their everyday life. This beckoning of the past was able to present them an opportunity to reflect on what had happened and also map ways to navigate their present circumstances. This is what is truly fascinating—when we project ourselves onto a map, when we map our spatial practices by identifying places tied to our activities and important aspects of our social and cultural identity, we place emphasis on how places aren’t simply locations. This introspective projection through drawing makes us realize how the territory shapes us, sculpts us, and transforms us.

## Conclusion

The first and most powerful strength of a map is its evocative power: it is a true dream machine in that, like cinema or literature, it evokes reality, serving as a receptacle for our desires to explore, our aspirations, but also as a projection of our imagination and our understanding of the reality. This evocative power naturally serves all political, social, and economic actors who find in cartography an effective tool to communicate and illustrate our relationship with space. It allows those in power to reinforce their positions and strengthen their control over the territories and populations they govern (in the best cases) or alienate (in the worst cases). For representatives of civil society and social actors, a critical approach to cartography enables the deconstruction of conventional representations of power, offering a way to visualize the world by making visible spatial and social inequalities and injustices. This form of cartography thus serves as a tool for struggles and civic movements that, in a quest for emancipation, strive to restore a sense of sovereignty to citizens over their living spaces. Thus, through cartography as an expression and representation of the world, both “power” and “counter-power” manifest themselves. This is why the map is fundamentally a political object (or weapon), which can be used to subjugate, impose, dominate, or manipulate—or, to resist and denounce.

Both experimental cartographers and critical social science researchers working with cartography have explored and refashioned the power-laden dream machine potential of maps. Thus far, however, there has been little cross-fertilization between experimental cartography and UPE. The purpose of this article has been to take a step toward fomenting this cross-fertilization. The experiential mapping practice we discuss, like other embodied methodologies, “involves the body as both its maker and researcher” (Solomon and Kaika, 2024: 1451), offering one *methodological* direction for developing a more embodied UPE. This not only brings often marginalized bodily knowledges to the forefront but emphasizes the embodied formation of political subjectivities that create capacities for action (Doshi, 2017).

The map has another interesting ability: it offers an immediate, comprehensive view of vast territories and a multitude of elements at a single glance—something impossible for a human being standing within those territories or even from an airplane to see. Its power lies in its ability to unapologetically reveal what usually remains invisible. Beyond representing a concealed reality, beyond what is strictly factual, one can also grasp the extraordinary power of cartographic expression in its ability to depict, literally to “draw,” the contours of our own emotions and territorial perceptions. By fully engaging our senses, we can capture and translate into drawings the way we experience space. This is the essence of experiential cartography.

We offer experiential cartography as a practice of (collectively) inhabiting, mapping, encoding and decoding, making meaning of, and intervening into everyday political ecologies. Our idea is that the poesis of experiential cartography is a methodology that involves simultaneously mapping embodied spatial practice/how everyday ecologies are perceived, creating embodied representations of space/alternative conceptions of everyday ecologies, and cultivating embodied spaces of representation/new meanings and spatial symbols from everyday life. The application of the approaches and protocols we developed in sensory mapping as part of the research conducted within the Embodied Ecologies project led to the emergence of what could be called “synthetic illustrations.” These offered immediate, easily understandable visual representations of subtle and complex issues, making visible aspects of reality often buried in the shadows of qualitative or quantitative data. Moreover, the very act of mapping feelings helped participants overcome psychological barriers, allowing them to better understand the reasons behind certain spatial practices, as well as their conditions and positions as social beings within the spaces they were mapping. This makes the tool a kind of complementary revealer in ethnographic studies, where the cartographic image can now engage in dialogue with the text to bring to light previously overlooked issues, processes, or trends. Privitera (2024: 46) similarly argues that “mapping toxic legacies with frontline communities can spark critical reflections and potentially reveal alternative visions for the future.”

The examples we’ve presented are preliminary work done as part of the Embodied Ecologies project that the authors have analyzed more in-depth elsewhere (Cordero, 2025; Echague, 2025; Pauchano, 2025; Pelagio, 2025). Far from expressing the total extent of what experiential cartography might do for UPE, they offer a sampling of potential directions for bringing embodiment into UPE methodologically. As Lefebvre (1991) makes clear, spaces—and, for that matter, ecologies—are embodied and, in part, produced through bodily activities and experiences. Experiential cartography is one way that UPE scholars may pay greater attention to such bodily activities and situated knowledges. Scholars working in the political ecology of toxicity have widely called for valorizing the bodily knowledges of those most directly affected, but bodily knowledges have received less emphasis in UPE scholarship. Finally, one of the potentials of experiential cartography only hinted at in the examples collected here is the movement from individual experiences to larger-scale processes, structures, and power-relations. In the Embodied Ecologies project, we are working to make this move analytically, textually, and cartographically; still these efforts are not fully represented in this article. Drawing such relations will surely require a multimodal approach to research and writing, with experiential cartography working best as one mode among others. As we claim at the beginning of this article, it is precisely such heterodox assemblages of theories and methods that have long been UPE's strength.
